# Protein and Aging: Practicalities and Practice

**DOI:** 10.3390/nu17152461

**Published:** 2025-07-28

**Authors:** Stephanie Harris, Jessica DePalma, Hope Barkoukis

**Affiliations:** Department of Nutrition, School of Medicine—WG 48, Case Western Reserve University, 10900 Euclid Avenue, Cleveland, OH 44106, USA; stephanie.harris@case.edu (S.H.); jessica.depalma@case.edu (J.D.)

**Keywords:** dietary protein, older adults, aging, muscle protein synthesis, sarcopenia, immunosenescence, chronic kidney disease, animal and plant protein, pre-sleep protein feeds, protein intake distribution

## Abstract

Dietary protein is an essential macronutrient derived from both plant and animal sources required for muscle building, immune function, and wound healing. However, in the United States, protein consumption worsens as individuals age, with 30% of men and 50% of women over 71 consuming inadequate dietary protein due to a variety of factors, including changes in gut function, loss of appetite, tooth loss, financial concerns, and social isolation. The aim of this review is to underscore the need for increased protein requirements in aging populations, highlight potential barriers, synthesize these protein requirements, and also recommend strategies to meet these increased protein needs. Achieving adequate protein status, especially when facing chronic or acute health concerns, is essential to promote muscle and bone strength (because aging is associated with significant decreases in postprandial muscle protein synthesis), to support immune health (due to immunosenescence), and to maintain a good quality of life. For older adults, the literature suggests that a dietary protein intake of at least 1.0–1.2 g/kg/day is required in healthy, aging populations, and intakes of 1.2–1.5 g/kg/day are necessary for those with chronic or acute conditions. These protein intake recommendations can increase to 2.0 g/kg/day in more severe cases of illness, malnutrition, and chronic conditions. The reviewed literature also suggests that evenly balanced protein distributions of 25–30 g of dietary protein (0.4 g/kg) per meal from animal and plant protein sources alike are sufficient to maximize muscle protein synthesis (MPS) rates in older populations. Additionally, pre-sleep protein feeds of 40 g/night may be another strategy to improve daily MPS and amino acid utilization.

## 1. Introduction

Aging is associated with complex physiological and psychosocial changes that dictate how older adults engage and interact in society [1]. These changes include sarcopenia, osteoporosis, poor digestion and absorption, neurological impairments, immunosenescence, chronic wounds, and worsened visual and auditory acuity [1,2,3,4]. These physiological changes and resulting disabilities are significant barriers to older adults maintaining their quality of life, contributing to 37% of older US adults (50–80 years old) experiencing loneliness and 34% experiencing social isolation, both of which can further increase chronic disease risk [5]. To promote health and well-being among the older population, it is important to consider how their dietary intake contributes to their aging experience. As we age, our macronutrient and micronutrient requirements must be adapted to the changes that come with each new stage of life [2,6,7,8]. This is especially important among the aging, considering one of the hallmarks of aging is the malabsorption of essential macronutrients and some micronutrients due to reduced saliva, decreased stomach acid production, decreased gastrointestinal motility, psychological factors, dysphagia, slower rates of gastric emptying contributing to elevated blood glucose and poor dentition [1,9,10,11,12]. All of these factors can make it difficult for aging populations to receive adequate caloric, macronutrient, and micronutrient intake, thereby contributing to muscle and bone loss, decreased immune function, and increased chronic disease risk [6,10]. Accounting for some of these changes, in their formal recommendations, the Dietary Guidelines for Americans (DGAs) identify specific nutrient deficiencies of concern in older Americans, including protein [2].

Dietary protein, found in meats, seafood, nuts, seeds, and dairy products, is one macronutrient that could help combat the adverse components and chronic diseases that are common during aging [2,13,14,15]. Older populations are at an increased risk for conditions such as sarcopenia and osteoporosis, characterized by losses in physical strength and functioning [7,8]. Research demonstrates that increased protein intake can slow the rate of lean muscle mass loss, known as sarcopenia, thereby contributing to less compromised muscle strength and function over time in a population characterized by decreased postprandial muscle protein synthesis (MPS) [7,16,17]. Muscle loss is a significant concern among the aging because all-cause mortality and death from cancer are inversely associated with muscular strength, highlighting the essentiality of muscle strength and function maintenance in older populations [17,18]. Moreover, decreased bone mineral density and osteoporosis risk are also associated with aging and poor physical functioning [7]. Improving muscular strength and increased dietary protein intake, combined with sufficient vitamin D and calcium intake, can help support optimal bone health through bone-loading and can improve bone mineralization through greater calcium absorption [7]. Moreover, in the case of illness and chronic disease, increased protein intakes are positively associated with cardiovascular health, wound healing, and recovery time, highlighting the need for adequate protein intake in populations suffering from chronic and/or inflammatory conditions to support immune health [4,8,19]. Immune health is heavily modulated by dietary protein intake, as amino acids such as arginine, glutamine, and cysteine are required to activate and proliferate immune system cell lines to combat illness and infection [19,20,21]. Furthermore, recent research demonstrates that chronic illness outcomes can be modulated by dietary intake, with one of the most significant effects of note being improvements in blood glucose control [22,23,24].

Currently, in the United States, men over 60 are, on average, consuming the recommended amount of daily protein; however, women fall below their recommended intake ranges [2]. The outlook on protein consumption worsens as individuals age; 30% of men and 50% of women over 71 consume inadequate protein [2,3]. Dietary protein consumption could play a significant role in helping older Americans maintain their autonomy and live full, independent, and healthy lives [13,14,15]. Thus, it is essential to have an understanding of the limitations and strategies required to ensure that aging Americans (ages 65 and older) are meeting adequate dietary protein intake levels. This review aims to underscore the relationship between protein intake and aging, focusing on muscle and bone health, immune function, and chronic disease risk, along with highlighting strategies to meet the most current recommendations.

### 1.1. Article Selection

Articles were selected from a date range of 1980 to 2025, with 81 of the articles dated within the last five years (2020 to 2025). Searches were conducted through PubMed and search terms included the following MeSH terms: dietary protein AND aging; dietary protein AND muscle protein synthesis; muscle protein synthesis; dietary protein AND anabolic resistance; anabolic resistance; leucine AND aging; leucine AND muscle protein synthesis; dietary protein AND chronic kidney disease; dietary protein AND immune health; dietary protein AND immunosenescence; immunosenescence; diet AND aging; malnutrition AND aging. Additionally, reference lists of papers included were also used to locate sources. The cited studies included those with systematic and narrative review designs, randomized controlled trials, clinical guidelines, and observational study designs.

### 1.2. Current Recommendations

#### 1.2.1. Dietary Protein Intake for Older Adults

Currently, the Recommended Dietary Allowance (RDA) for protein in adults is 0.8 g/kg of body weight; however, it is worth noting that minimal adverse effects are observed at higher intakes to promote improved MPS [17,25]. Therefore, in older adults at an increased risk of muscle and strength loss, increasing optimal intakes to above the RDA may support improvements in protein-related physiological functions [6,8,10,17]. This intake range is between 1.0 g and 1.2 g per kilogram of body weight for improved MPS and maintenance of lean body mass [6,8,10,17,26,27,28,29,30]. Additionally, due to increased protein needs to combat illness, the research also indicates that for older adults with malnutrition, acute illness, chronic disease, risks for frailty, and/or other inflammatory conditions, dietary protein intake should be increased to 1.2–1.5 g/kg of body weight, with more severe cases requiring up to 2.0 g/kg of body weight to support immune activity [4,6,7,8,27,28,31,32,33,34]. To meet these protein needs, research suggests that it is important for older adults to have a balanced distribution of protein intake per meal, representing roughly 25–30 g of protein, to maximize MPS [26,27,28,29,30,34,35,36]. Strategies to achieve these intake levels are discussed later in this review.

In addition to the Dietary Guidelines for Americans’ (DGAs) recommendations, the European Food and Safety Authority recommends a Population Reference Intake of 0.83 g of protein/kg of bodyweight/day among all adults (similar to the DGAs) [37]. The European Society for Clinical Nutrition and Metabolism (ESPEN), on the other hand, specifies the need for more nuanced recommendations for older adults (1.0–1.2 g/kg/day for healthy older people, and 1.2–1.5 g/kg/day for those with acute or chronic illnesses) [29].

#### 1.2.2. Leucine Intake for Muscle Protein Synthesis

While consumption of all of the essential amino acids is necessary to achieve adequate protein status, research has indicated that leucine consumption, in particular, may be especially important to promote MPS [26,29,30,35]. Previous research has alluded to a connection between leucine supplementation and the activation of the mammalian target rapamycin pathway (mTOR), leading to increased MPS, even when total protein intake was logged as below 1.0 g/kg/day (0.8 g/kg/day) [26,29,30]. Leucine is a key activator of the mTOR pathway, triggering mTOR to initiate MPS following a meal; however, in older populations, there is a decrease in the activation of mTOR and MPS in response to protein intake that can be described as “anabolic resistance”, suggesting that greater amounts of leucine, in particular, may be required to promote greater MPS and decreased proteolysis due to the amino acid’s critical role as both a substrate and a signal [8,26,29,30,34,35,36,38,39,40,41]. In Kuczmarski et al., intake of leucine and other branched-chain amino acids (BCAAs) improved MPS as indicated by the positive association between the ratio of handgrip strength and BMI to intake of BCAAs [35].

To adjust for the limitations of decreased postprandial MPS in aging, some studies claim that older adults should consume 2.8–3 g of leucine with each meal (78.5 mg/kg/day compared to the current RDA of 34 mg/kg/day), which can be achieved through high-quality protein dietary intake or oral nutrition supplementation [34,36,40,41,42,43].

### 1.3. Nuances of Protein Recommendations for Aging

#### 1.3.1. Anabolic Resistance

Anabolic growth processes involve a complex signaling environment whereby insulin and insulin-like growth factor (IGF1) are critical in the body’s responses to growth signals [44]. Once insulin and IGF1 are activated, a metabolic cascade ensues resulting in activation of phosphoinositide 3 kinase (PI3K), and subsequently Akt (a serine/threonine-protein kinase also known as protein kinase B) [44]. There are multiple targets for activated Akt to modulate growth and metabolic processes through. Activated Akt also is an effector for a complex known as mTOR (mechanistic target of rapamycin), which is a protein kinase [44]. Detailed discussion of mTOR metabolism is beyond the scope of this review, except to state that mTOR regulates cellular responses and forms two complexes: MTORC1 and MTORC2. The net result from activation of mTOR is an increased synthesis of nucleotides, lipids, and proteins [36,44,45].

Anabolic resistance of aging refers to the phenomena of a blunted physiologic response to the anabolic stimulus of amino acids and exercise on muscle protein synthesis [26,29,30,34,36,40,41,43,46,47,48,49,50]. Numerous mechanisms have been identified as components of the etiology of this anabolic resistance of aging [36,44,45]. Some of these key factors are not limited to, but include a blunted Akt phosphorylation response, dysregulation of PI3K/Akt/mTOR signaling, increased presence of proinflammatory cytokines, increases in oxidative stress associated with senescence, altered enzymatic functioning, and obesity [36,44,45]. This resistance to stimulation by plasma amino acids has detrimental effects, as postprandial MPS rates are significantly lower in older male populations than in younger men (a 16% decrease in MPS rates), contributing to the loss of muscle strength and mass observed in sarcopenia and suggesting that older adults may need to increase their protein intake to adjust for the decreased MPS stimulation [7,16,17,28,32,33,36,38,47,48,51]. It is worth noting that this aforementioned study did not observe a significant difference in the post-absorptive MPS rates of older and younger populations, suggesting that post-prandial MPS and aminoacidemia responses are more directly altered in the aging process than post-absorptive MPS [36,48,52]. However, a study evaluating young and old adult men completing unilateral leg exercises following an overnight fast (post-absorptive) state contradicts this idea by finding that MPS was only elevated in the young participants’ exercised leg relative to their non-exercised leg; there was no significant difference between the legs of the older participants, suggesting that there may be age-related difference in post-absorptive MPS rates [53].

Aragon et al. and Tezze et al. suggested similar protein intakes of 0.4 g/kg–0.6 g/kg and 0.4 g/kg per meal, respectively, for aging individuals to counter the effects of anabolic resistance [28,49]. In one study, compared to younger adults, older adults required almost double the amount of protein per meal (0.4 g/kg compared to 0.24 g/kg) to maximize MPS rates, further demonstrating the effects of anabolic resistance on post-prandial MPS in aging populations and the need for nuanced protein recommendations [49,51].

#### 1.3.2. Anabolic Resistance and Loss of Muscle Mass and Strength (Sarcopenia)

Adequate dietary protein is required to activate the mTOR pathway and stimulate MPS, which occurs 2–5 h postprandially [32,38,48]; however, MPS is decreased in older populations due to decreased activation of mTOR by ingested protein, leading to an increased risk of sarcopenia [7,16,17,32,33,38,47]. The counter of MPS is muscle protein breakdown (MPB), or proteolysis; however, this process is not as dependent on protein ingestion, suggesting that post-prandial MPS is a better target for dietary intervention to decrease sarcopenia risk [36]. While there is some debate over the exact clinical definition of sarcopenia, the condition is characterized by a state of muscle mass and strength loss contributing to poor physical performance, which is associated with an elevated risk for fractures [3,7,16,17,46]. According to Chen et al., the condition is most common in adults aged 65+ and can contribute to significant decreases in quality of life due to functional limitations in day-to-day life and an increased risk for falls and death [46,47,54,55,56]. Sarcopenia’s contribution to increased fracture risk illustrates the positive correlation between sarcopenia and osteoporosis, another common aging condition characterized by low bone mineral density and an increased risk for fractures [46,54]. Yu et al.’s systematic review demonstrates that significant decreases in osteoporosis risk are associated with increases in appendicular skeletal muscle mass, and individuals with osteoporosis have a greater likelihood of also having sarcopenia [54]. This connection can be explained by the bone-loading and mechanical force that skeletal muscle mass inflicts on bone, promoting improved or maintained bone density [7,26,29,30].

In one study evaluating grip strength as an indicator of sarcopenia, researchers noted a positive association between the ratio of handgrip strength to BMI and essential amino acid (EAA) intake per kg of body weight, researchers also determined that adults over the age of 50 had decreased EAA consumption compared to younger adults [35]. Additionally, in another study evaluating middle-aged and aging participants, women who had the greatest dietary protein intake, higher than the RDA (0.8 g/kg/day), had a significantly decreased risk for sarcopenia (a 35–50% decrease in risk compared to women who fell into the lowest quartile of intake) [46]. In Moyama et al., researchers determined that protein intake and skeletal muscle mass index (SMI) were positively correlated in older adults with Type 2 diabetes who came to the hospital as outpatients [32]. Additionally, among inpatients, including older adults with acute and/or chronic conditions, another study demonstrated that intakes of 1.3–1.5 g/kg/day lead to significant positive changes in grip strength compared to participants receiving 1.0 g/kg/day of protein [33]. Peng et al. considered older adults with suboptimal protein intake (<1.0 g/kg/day) and determined that increasing intake to 24–30 g of protein via protein-enriched soup and weekly hour-long workouts led to significant improvements in grip strength and other markers of physical performance (the sit-to-stand test), highlighting the maintenance of muscle strength [27].

As demonstrated in the literature, poor dietary protein intake and decreased MPS stimulation due to anabolic resistance in older adults are associated with losses in muscle mass and strength, which is one of the hallmarks of sarcopenia, suggesting that improvements in dietary protein intake (between 1.0 to 1.5 g/kg/day depending on chronic/acute illness status) could help to support an improved quality of life and abated rates of muscle loss [27,32,33,35,46,47].

#### 1.3.3. Immune System Function and Immunosenescence

The process of normal aging, or senescence, is accompanied by a ubiquitous decline in overall immune system functioning, and this process is called immunosenescence. These alterations include changes in the function, composition, and quality of organ systems, immune cells, and cytokines [57]. Immunosenescence, which leads to chronic inflammation and an inability to recognize and combat new antigens, is a significant concern for the health of older populations, resulting in an increased susceptibility to infection and disease [57,58,59].

It has been well described that immunosenescence is secondary to a complex sequence of events with a multifactorial etiology [57]. The precise molecular mechanisms and degree to which each factor impacts the ultimate changes in systemic immune functioning observed with senescence is under active investigation [57,58,59,60,61]. Some of the broad areas of investigation include chronic inflammation, alterations in telomere functioning and length, and a senescence-associated secretory phenotype (SASP) [57]. This proinflammatory state includes alterations in chemokines, growth factors, inflammatory factors, and matrix proteases. SASP is well described as a ubiquitous characteristic of senescent cells. SASP is impactful in inducing this process of immunosenescence because it impacts a multiplicity of signal pathways, which induces systemic inflammation [57,58,59,60,61]. The term “inflammaging’ is universally observed with the normal aging process. It is characterized by a low grade, chronic state of inflammatory reactions [57,58,59,60,61]. These collective inflammatory reactions ultimately result in the metabolic milieu of immunosuppression [57,58,59,60,61].

Increased chronic inflammation, infection, and disease due to immunosenescence can directly impact the protein requirements of aging populations [4,6,7,8,27,28,31,32,33,34,40,41,43,57,58,59,60,61]. Additionally, a low protein status can also increase the aging’s risk of infection, poor vaccine responses, and cancer mortality, in part due to the specific roles amino acids play in activating immune cell proliferation, maintaining the appropriate cellular redox state and producing antibodies and cytokines [20,62,63,64]. Related to vaccine responses, which is of significant relevance given the recent COVID-19 pandemic, recent research demonstrates that the maintenance and proliferation of T cells may be negatively impacted by a low protein status [65,66,67]. Additionally, one mouse study noted a decrease in the frequency of CD4+ T cells and hepatic natural killer cells, while CD8+ T cells were unaffected—suggesting that different T cell types may be differently impacted [65]. More research on human subjects is needed [65]. Amino acids are required for immune system function, as efficient and rapid cell proliferation is necessary for the immune system to combat pathogens [68] For example, when CD8+ T cells are activated, there is an increase in the expression of amino acid transporters, especially glutamine transporters, to supply the nutrients for increased T cell proliferation [68,69]. Glutamine, leucine, isoleucine, and valine are all required for the changes in metabolic activity that are necessary for immune cells to proliferate and respond to pathogens; furthermore, glutathione (which is made up of cysteine, glutamine, and glycine) is needed to detox reactive oxygen species (ROS) in inflammatory immune environments, and production of this antioxidant is increased by T cell stimulation [20,68,70,71]. Glutathione has been shown to halt the proliferation of influenza, and nitric oxide made from arginine can help to destroy invading pathogens, further demonstrating the critical role that protein intake has on immune function [20,70].

#### 1.3.4. Renal Function and Health

Among older populations, especially those diagnosed with or at an increased risk of chronic kidney disease (CKD), there is concern that high protein intakes (1.2–2.0 g/kg/day) may lead to glomerular hyperfiltration and proteinuria [72,73]. Hyperfiltration can lead to kidney damage, and proteinuria is an important early sign of this damage; however, the research is somewhat conflicting, with some short-term studies noting a connection, and other long-term studies noting no significant associations [72,73,74].

CKD has a significant global disease burden, with over 800 million people diagnosed and 20% of adults aged 75 and older progressing from CKD to end-stage of renal disease [75] Traditionally, dietary protein intake restrictions (0.8 g/kg/day at most) have been a critical nutrition intervention employed by older patients with CKD to manage and slow disease progression [64,76,77,78,79]. Guidelines from the National Kidney Foundation’s Kidney Disease Quality Initiative (KDOQI) recommend an intake of 0.55–0.60 g/kg/day of dietary protein (or 0.28–0.43 g of dietary protein/kg/day supplemented with amino acid or keto acid analogs to make up the difference) for patients with CKD who are not currently on dialysis and without diabetes (recommendations for those with diabetes are slightly different) [78,79]. For CKD patients with diabetes who are not on dialysis, this recommendation increases to 0.6–0.8 g/kg/day of dietary protein [78,79]. A review by Hahn et al. noted that a very low protein intake (0.3 to 0.4 g/kg/day) likely helps to slow the progression of CKD to kidney failure and the need for dialysis among non-diabetic adults (ages ranging from 18 to 75, with most studies having a mean age between 35 and 65) with moderate-to-severe CKD and excluding those on peritoneal dialysis or hemodialysis, and those with kidney transplants [76]. In aging patients (median age of 75) with advanced Diabetic Kidney Disease and without any comorbidities such as heart failure, infection, liver cirrhosis, steroid use, and gastrointestinal disorders, low protein diets (0.6 g/kg/day) were associated with delays in kidney transplants and decreased proteinuria, suggesting that a low dietary protein intake may be able to support kidney function in this patient population [80]. Jhee et al. noted an increased risk for renal hyperfiltration and declines in kidney function associated with high protein intake among the subjects from the Korean Genome and Epidemiology Study cohort (ages 49–60 years old) without kidney disease, representing the general population [73].

Despite the research pointing to low-protein diets as effective managers of CKD risk and progression, other studies note that increased protein intake may be better for overall health. Carballo-Casla et al. noted decreases in all-cause mortality associated with higher protein intakes among older patients from the Study on Cardiovascular Health (ages 60 and older) and Nutrition and Frailty in Older Adults in Spain 1 and 2 cohorts (ages 65 and older) with CKD (excluding stages 4 and 5, along with those with kidney transplants), suggesting that while protein may lead to declines in kidney function, it is still essential for healthy aging and that increases in protein intake may be more beneficial than harmful for these populations [81]. Langsetmo et al. supported these findings, noting an increased risk of death with decreased protein intake among ambulatory men at least 65 years old [64]. Additionally, Moyoma et al. found that modest increases in protein intake (1.3 g/kg/day) did not affect the renal function of adults aged 75 and older who were hospitalized for cancer, fractures, urinary-tract infections, or pneumonia; this study excluded conditions such as psychiatric disorders, heart failure, severe diabetes complications, untreated cancer, and gastrointestinal reconstruction surgery, among others [33]. Moreover, the source of protein from the diet may also be of note, with research demonstrating that low-protein diets from plant-based sources may support chronic kidney disease management and contribute to a decreased risk of CKD [77,78].

#### 1.3.5. Other Considerations (Finances, Dentition, and Appetite)

Despite the positive impacts of increasing dietary protein intake among older adults, making dietary adjustments for this population poses a unique set of challenges. The cost of food items is a determining factor in food selection, so it is worth noting that a protein-rich diet is often more expensive than a diet lacking sufficient protein for older adults [82,83,84]. According to data from the USDA, protein foods (not considering dairy and mixed dishes) make up 24% of food costs among American adults; when dairy and mixed dishes were considered, this percentage ballooned up to 52% [83]. Additionally, more expensive and healthier (scoring higher on the Healthy Eating Index) diets are associated with older populations [85]. Nevertheless, there are ways to adapt to these cost challenges. Rautakallio-Järvinen et al. determined that a protein-rich diet (containing one home-delivered meal and snacks that are high in protein) was more cost-effective than relying on home-delivered meals alone [82]. Additionally, Papanikolaou et al. determined that eggs were a cost-effective food to support daily dietary protein intake [83]. This research suggests that older adults can maximize their spending on dietary protein by using a combination of meats and other sources such as whole grain bread, eggs, and dairy products, either as a part of a meal or a snack throughout the day [82,83].

Additionally, other significant challenges affecting dietary protein intake among older adults include appetite loss and dentition concerns [86,87]. Among older adults living in the community, appetite loss and decreased intake are common, experienced by 15–30% of this population, and are attributed to changes in dentition, gut function, vision, taste, smell, illness, physical activity, loneliness, mental health, and medications [86,88]. Appetite loss can directly impact protein consumption, with 20% of older Americans not reaching the dietary protein RDA of 0.8 g/kg/day due in part to the satiating nature of protein-rich foods, which can make balancing protein needs with overall energy needs challenging [87]. The same below-needs protein habits were observed in older Brazilian populations, who consumed an average of 72 g of protein/day, significantly lower than the requirement (77 g of protein/day) [89]. Regarding dentition and oral health, poor dietary intake can contribute to poor oral health and vice versa [90]. Oral health is of a significant concern for the dietary intake of older Americans because, according to the CDC, roughly 17% of Americans aged 65+ have lost all of their teeth [91,92]. Poor oral health (specifically tooth loss, regardless of whether dentures are used) is associated with advancing frailty in older populations, and one plausible culprit for this connection is the resulting changes in dietary intake, including decreases in protein intake, due to tooth loss and difficulty swallowing [93,94,95,96]. Tooth loss and other oral health concerns can make achieving adequate nutritional status difficult; therefore, dentition status is an important factor to consider in dietary protein recommendations for older adults [93,94,95,96].

### 1.4. Supporting Protein Needs During Aging

#### 1.4.1. Daily Meal Distribution, Timing, and Patterns

Among older adults, protein consumption is skewed toward later in the day, with breakfast having the lowest intake; however, research indicates that this common practice may be detrimental to the maintenance of muscle mass, though the mechanism of how is still debated, and could lead to a decrease in total protein intake [25,28,97,98,99,100,101,102,103]. In one systematic review, among healthy adults, MPS rates were increased by 25% when protein intake was balanced across three meals compared to the same protein intake skewed towards later in the day [25]. Nevertheless, another review observed an association between even protein intake and increased muscle mass, but not MPS, with Justesen et al. also noting no difference in MPS rates between protein distributions [104,105]. In a study of healthy aging individuals, increased absorption rates of amino acids, which are critical for MPS, and decreased protein breakdown rates were observed in the group following an even protein distribution compared to those following a skewed distribution plan (30% at each meal vs. 15%, 60%, and 60%, with snacks provided for the remaining 5% in both groups) [97] By decreasing protein breakdown, a positive protein net-balance can be achieved to decrease both muscle mass loss and sarcopenia risk [97]. Together, these findings indicate that there is still some debate as to the mechanism of how an even protein meal distribution affects muscle mass maintenance, whether it be by increasing amino acid absorption or muscle protein synthesis rates [25,28,97,98,99,100,101,102,103].

A balanced protein distribution not only affects absorption and biological mechanisms, but also total protein dietary intake, with Verreijen et al. finding that increased protein intake at breakfast and lunch promotes a higher total protein intake per day among older men and women [98]. Given that decreased appetite is of critical concern for older adults and protein foods are often very satiating, optimizing daily protein intake by spreading protein intake out throughout the day is an important step to reaching intake goals [86,87,88,98,106]. Hiol et al. further supported this point by noting a positive association between muscle strength and the frequency of meals containing 0.4 g/kg (25–30 g per meal) [100]. In a recent study evaluating older adults, only 1% of participants ate more than 20 g protein/meal for each meal, demonstrating the need for sound nutrition guidance from healthcare professionals [101].

While the mechanism by which evenly distributed protein intake impacts protein utilization and MPS rates remains somewhat elusive, balanced protein distributions across meals may be a useful dietary practice to promote increased total protein intake and adapt to changes in appetite and fullness [25,28,86,87,88,97,98,99,100,101,102].

Regarding the amount per meal, the maximum activation of MPS occurs with a protein intake of 0.4 g/kg of body weight per meal, which is roughly 25–30 g of dietary protein per meal, for four meals (breakfast, lunch, snack, and dinner) [26,27,28,29,30,34,35,36,40,41,42,43,51,107]. However, a recent study suggests that the amount of protein ingested versus the spacing of this protein intake may be more important, with one protein-full meal being enough to stimulate adequate MPS [50,108]. Trommelen et al. observed a greater, sustained anabolic response in young, healthy adults over a period of 12 h to 100 g of protein in one meal versus 25 g [50]. Nevertheless, these findings cannot and should not be widely applied to older adults due to the marked decrease in MPS rates and the increased risk for CKD and other renal health conditions that can be affected by protein intake in this population [7,16,17,32,33,38,47,72,73]. Spacing protein intake throughout a few meals, with an even distribution of protein intake, can maximize protein intake and allow for better satiety management and adjustments for decreased appetite, suggesting that a balanced protein intake distribution is the best course of action for older adults [25,28,86,87,88,97,98,99,100,101,102].

Another area of interest regarding protein intake patterns is pre-sleep, late-night protein intake, which may further support improvements in MPS [109,110,111,112,113]. Holwerda et al. found that pre-sleep ingestion of 40 g of casein in combination with resistance exercise enhanced MPS rates during rest compared to controls and groups that took the pre-sleep protein without exercise [109]. These findings are consistent with Trommelen et al., who noted that 40 g of protein ingested before sleep was sufficient to activate overnight MPS following resistance exercise, a process that is typically limited by the decreased availability of amino acids during rest [110,112,113]. Furthermore, Weijzen et al. found that, in hospitalized adults, pre-sleep protein intake supports increases in overall daily energy and protein intake [111]. Taken together, these studies suggest that the ingestion of 40 g of pre-sleep protein, coming from sources such as casein or whey supplements or animal-sourced protein concentrates, could not only support meeting protein intake targets but may also promote increased overnight MPS in aging populations [109,110,111,112,113].

#### 1.4.2. Choosing the Right Sources (Plant vs. Animal)

Protein can be sourced from both plant- and animal-based foods; however, only animal proteins are complete proteins, meaning that they contain a balanced array of all of the essential amino acids [25,47]. Complementary pairings of plant-based proteins can be employed to consume all of the EAAs and prevent malnutrition, but it is worth noting that eating meat can significantly decrease the volume of plant-based foods necessary to meet dietary protein recommendations, which could be necessary for older adults with poor appetites and a preference for smaller meals [25,47,114,115]. Additionally, animal proteins typically have an increased bioavailability and higher amounts of leucine, which is necessary for MPS that is already dampened in older adults due to anabolic resistance [26,29,30,34,35,36,38,39,40,41,43,116,117,118]. Pinckaers et al. found that compared to a vegan meal, older adults who consumed an omnivorous meal with beef had roughly 47% higher rates of postprandial MPS, with a 25% higher increase in postprandial plasma leucine concentrations [119]. Additionally, in one study evaluating the implications of animal protein and plant protein consumption on grip strength in aging populations, plant protein intake had no protective effect on grip strength maintenance [47]. Oppositely, animal protein intake was positively associated with functional status and improved grip strength maintenance, especially among the sedentary study participants and those with low skeletal muscle mass [47]. McLean et al. noted similar findings with animal protein intake being significantly associated with grip strength maintenance in adults aged 60 and older, while plant protein intake’s association with grip strength maintenance was insignificant [120]. Among older women, animal protein intake is significantly associated with grip strength maintenance [116]. Further supporting these results, Lim et al. determined that animal protein was preferred for lean mass percentage, but this effect was more substantial among younger adults and was not observed regarding differences in absolute lean mass or strength between protein sources [117,121].

Despite the benefits of animal protein on MPS rates and grip strength, researchers note that animal-based protein sources are not an option for older Americans who follow a vegan or vegetarian diet; additionally, plant-based protein sources have numerous benefits for aging populations that should not be ignored [38,47,77,115]. Ardisson et al. noted that for the prevention of poor aging, plant protein, which has less saturated fat and is more anti-inflammatory compared to animal protein sources, is best among healthy, middle-aged females [38,46,77,117]. Higher intake of plant protein was associated with improved maintenance of physical performance in older, female adults, but these findings are contradicted by another study that noted a significant association between animal protein intake and grip strength maintenance [122]. These findings not only demonstrate that plant protein sources may be able to adequately support healthy aging, but also that there may be some sex-based differences in the degree to which plant proteins can support healthy aging [38,46,77,116,117,122]. Additionally, higher intakes of red and processed meats are associated with the incidence of CKD and progression of the disease, whereas plant-based protein options may be protective against CKD and kidney failure (replacing one serving of red meat with plant-based protein leads to a 50.4% drop in kidney failure risk) [77]. In regard to physical performance and muscle (grip) strength, plant proteins from legumes, nuts, and seeds were associated with higher grip strengths among older adults, but other plant protein sources (i.e., cereals, starches, vegetables, and products made from these) did not exhibit the same associations [116]. Similarly, Du et al. found that, when confounding variables were controlled for, plant-based diets with high dietary diversity scores were associated with a lower risk for sarcopenia; however, this same association was not found for plant-based diets that lack legumes and nuts, suggesting that the type of plant protein chosen is important [116,123].

When selecting sources for protein, diversifying options to include a balanced selection of animal- and plant-based foods may be the best option for omnivorous patients [38,46,47,77,116,117,118,119,121,123,124]. To maximize the health benefits of animal proteins that can increase MPS rates and have high EAA bioavailabilities, individuals should seek to limit their consumption of pro-inflammatory and high-saturated fat animal products such as red meat and processed meats while also increasing their intake of anti-inflammatory plant-based protein sources such as nuts, legumes, and seeds that have demonstrated positive associations with physical performance and sarcopenia risk.

## 2. Conclusions

An adequate dietary protein intake of at least 1.0–1.2 g/kg/day among healthy older adults and intakes of 1.2–1.5 g/kg/day for those with chronic or acute conditions is of critical importance to promote muscle and bone strength, immune health, and overall quality of life. However, due to factors such as loss of appetite, anabolic resistance, financial concerns, and dentition, achieving adequate dietary protein intake poses a unique challenge among this population. The reviewed literature suggests that evenly balanced protein distributions of 25–30 g of dietary protein (0.4 g/kg of bodyweight) per meal can maximize MPS in aging populations. Additionally, pre-sleep protein feeds of 40 g/night may be another strategy to improve daily MPS rates and amino acid utilization. Both animal and plant protein sources can be used to meet these goals, with animal proteins being best for MPS and plant proteins having strong anti-inflammatory effects and a lower saturated fat content. Patients should focus on integrating legumes, nuts, and seeds as main sources of plant protein and, for those who eat meat, limiting red and processed meats in the diet to maximize the benefits of each source and decrease their risk for cardiovascular disease, cancer, and other chronic inflammatory conditions. For patients with or at risk of chronic kidney disease, a lower protein diet may be advantageous; however, these diets should only be undertaken per the recommendation and guidance of a physician or registered dietitian nutritionist. Current gaps in knowledge include an understanding of the mechanism and extent to which dietary protein intake affects vaccine responses in older adults, along with more research into the best source of protein for pre-sleep protein feeds. Additionally, more research regarding the specific protein requirements for various disease states that are common in aging, such as cardiovascular disease, stroke, cancer, and osteoporosis, to create streamlined, nuanced guidelines for older adults with these conditions is needed.

## Abbreviations

The following abbreviations are used in the manuscript:

MPS	muscle protein synthesis
DGA	dietary guidelines for Americans
BCAA	branched-chain amino acids
MBP	muscle protein breakdown
EAA	essential amino acid
SMI	skeletal muscle index
ROS	reactive oxygen species
CKD	chronic kidney disease

## References

[1] Dziechciaż M., Filip R. (2014). Biological psychological and social determinants of old age: Bio-psycho-social aspects of human aging. Ann. Agric. Environ. Med..

[2] U.S. Department of Agriculture (2020). Dietary Guidelines for Americans, 2020–2025.

[3] Lees M., Carson B. (2020). The Potential Role of Fish-Derived Protein Hydrolysates on Metabolic Health, Skeletal Muscle Mass and Function in Ageing. Nutrients.

[4] Vranešić Bender D., Krznarić Ž. (2020). Nutritional issues and considerations in the elderly: An update. Croat. Med. J..

[5] Gerlach L.B., Solway E.S., Malani P.N. (2024). Social Isolation and Loneliness in Older Adults. JAMA.

[6] Volkert D., Beck A.M., Cederholm T., Cruz-Jentoft A., Goisser S., Hooper L., Kiesswetter E., Maggio M., Raynaud-Simon A., Sieber C.C. (2019). ESPEN guideline on clinical nutrition and hydration in geriatrics. Clin. Nutr..

[7] Rizzoli R., Stevenson J.C., Bauer J.M., Van Loon L.J.C., Walrand S., Kanis J.A., Cooper C., Brandi M.-L., Diez-Perez A., Reginster J.-Y. (2014). The role of dietary protein and vitamin D in maintaining musculoskeletal health in postmenopausal women: A consensus statement from the European Society for Clinical and Economic Aspects of Osteoporosis and Osteoarthritis (ESCEO). Maturitas.

[8] Bauer J., Biolo G., Cederholm T., Cesari M., Cruz-Jentoft A.J., Morley J.E., Phillips S., Sieber C., Stehle P., Teta D. (2013). Evidence-Based Recommendations for Optimal Dietary Protein Intake in Older People: A Position Paper from the PROT-AGE Study Group. J. Am. Med. Dir. Assoc..

[9] Woudstra T., Thomson A.B.R. (2002). Nutrient absorption and intestinal adaptation with ageing. Best Pract. Res. Clin. Gastroenterol..

[10] Borkent J., Manders M., Nijhof A., Wijker L., Feskens E., Naumann E., De Van Der Schueren M. (2023). Too low protein and energy intake in nursing home residents. Nutrition.

[11] Roberts S.B., Rosenberg I. (2006). Nutrition and Aging: Changes in the Regulation of Energy Metabolism with Aging. Physiol. Rev..

[12] Evans M.A., Triggs E.J., Cheung M., Broe G.A., Creasey H. (1981). Gastric Emptying Rate in the Elderly: Implications for Drug Therapy. J. Am. Geriatr. Soc..

[13] Coelho-Júnior H.J., Calvani R., Tosato M., Landi F., Picca A., Marzetti E. (2022). Protein intake and physical function in older adults: A systematic review and meta-analysis. Ageing Res. Rev..

[14] Hu F.B. (2024). Diet strategies for promoting healthy aging and longevity: An epidemiological perspective. J. Intern. Med..

[15] Muth A.-K., Park S.Q. (2021). The impact of dietary macronutrient intake on cognitive function and the brain. Clin. Nutr..

[16] Houston D.K., Nicklas B.J., Ding J., Harris T.B., Tylavsky F.A., Newman A.B., Lee J.S., Sahyoun N.R., Visser M., Kritchevsky S.B. (2008). Dietary protein intake is associated with lean mass change in older, community-dwelling adults: The Health, Aging, and Body Composition (Health ABC) Study. Am. J. Clin. Nutr..

[17] Wolfe R.R. (2012). The role of dietary protein in optimizing muscle mass, function and health outcomes in older individuals. Br. J. Nutr..

[18] Ruiz J.R., Sui X., Lobelo F., Morrow J.R., Jackson A.W., Sjostrom M., Blair S.N. (2008). Association between muscular strength and mortality in men: Prospective cohort study. BMJ.

[19] Cawood A.L., Elia M., Stratton R.J. (2012). Systematic review and meta-analysis of the effects of high protein oral nutritional supplements. Ageing Res. Rev..

[20] Li P., Yin Y.-L., Li D., Woo Kim S., Wu G. (2007). Amino acids and immune function. Br. J. Nutr..

[21] Lesourd B. (1997). Nutrition and immunity in the elderly: Modification of immune responses with nutritional treatments. Am. J. Clin. Nutr..

[22] Shahnaz T., Fawole A.O., Adeyanju A.A., Onuh J.O. (2024). Food Proteins as Functional Ingredients in the Management of Chronic Diseases: A Concise Review. Nutrients.

[23] Gannon M.C., Nuttall F.Q., Saeed A., Jordan K., Hoover H. (2003). An increase in dietary protein improves the blood glucose response in persons with type 2 diabetes. Am. J. Clin. Nutr..

[24] Samkani A., Skytte M.J., Kandel D., Kjaer S., Astrup A., Deacon C.F., Holst J.J., Madsbad S., Rehfeld J.F., Haugaard S.B. (2018). A carbohydrate-reduced high-protein diet acutely decreases postprandial and diurnal glucose excursions in type 2 diabetes patients. Br. J. Nutr..

[25] Wu G. (2016). Dietary protein intake and human health. Food Funct..

[26] Nowson C., O’Connell S. (2015). Protein Requirements and Recommendations for Older People: A Review. Nutrients.

[27] Peng L., Lin M., Tseng S., Yen K., Lee H., Hsiao F., Chen L. (2024). Protein-enriched soup and weekly exercise improve muscle health: A randomized trial in mid-to-old age with inadequate protein intake. J. Cachexia Sarcopenia Muscle.

[28] Aragon A.A., Tipton K.D., Schoenfeld B.J. (2023). Age-related muscle anabolic resistance: Inevitable or preventable?. Nutr. Rev..

[29] Deutz N.E.P., Bauer J.M., Barazzoni R., Biolo G., Boirie Y., Bosy-Westphal A., Cederholm T., Cruz-Jentoft A., Krznariç Z., Nair K.S. (2014). Protein intake and exercise for optimal muscle function with aging: Recommendations from the ESPEN Expert Group. Clin. Nutr..

[30] Lee S.Y., Lee H.J., Lim J.-Y. (2022). Effects of leucine-rich protein supplements in older adults with sarcopenia: A systematic review and meta-analysis of randomized controlled trials. Arch. Gerontol. Geriatr..

[31] McClave S.A., Martindale R.G., Vanek V.W., McCarthy M., Roberts P., Taylor B., Ochoa J.B., Napolitano L., Cresci G., A.S.P.E.N. Board of Directors (2009). Guidelines for the Provision and Assessment of Nutrition Support Therapy in the Adult Critically Ill Patient: Society of Critical Care Medicine (SCCM) and American Society for Parenteral and Enteral Nutrition (A.S.P.E.N.). J. Parenter. Enter. Nutr..

[32] Moyama S., Yamazaki Y., Takahashi T., Makabe N., Hamamoto Y., Kurose T., Yamada Y., Kuwata H., Seino Y. (2025). Dietary Protein Intake Is a Determining Factor for Skeletal Muscle Mass in Japanese Older People with Type 2 Diabetes: A Cross-Sectional Study. Nutrients.

[33] Moyama S., Yamada Y., Makabe N., Fujita H., Araki A., Suzuki A., Seino Y., Shide K., Kimura K., Murotani K. (2023). Efficacy and Safety of 6-Month High Dietary Protein Intake in Hospitalized Adults Aged 75 or Older at Nutritional Risk: An Exploratory, Randomized, Controlled Study. Nutrients.

[34] Cereda E., Pisati R., Rondanelli M., Caccialanza R. (2022). Whey Protein, Leucine- and Vitamin-D-Enriched Oral Nutritional Supplementation for the Treatment of Sarcopenia. Nutrients.

[35] Kuczmarski M.F., Beydoun M.A., Zonderman A.B., Evans M.K. (2022). Intakes of Total and Branched-Chain Essential Amino Acids are Positively Associated with Handgrip Strength in African American and White Urban Younger and Older Adults. J. Nutr. Gerontol. Geriatr..

[36] Paulussen K.J.M., McKenna C.F., Beals J.W., Wilund K.R., Salvador A.F., Burd N.A. (2021). Anabolic Resistance of Muscle Protein Turnover Comes in Various Shapes and Sizes. Front. Nutr..

[37] EFSA Panel on Dietetic Products (2012). Scientific opinion on dietary reference values for protein. EFSA J..

[38] Ardisson Korat A.V., Shea M.K., Jacques P.F., Sebastiani P., Wang M., Eliassen A.H., Willett W.C., Sun Q. (2024). Dietary protein intake in midlife in relation to healthy aging—Results from the prospective Nurses’ Health Study cohort. Am. J. Clin. Nutr..

[39] Rehman S.U., Ali R., Zhang H., Zafar M.H., Wang M. (2023). Research progress in the role and mechanism of Leucine in regulating animal growth and development. Front. Physiol..

[40] Szwiega S., Pencharz P.B., Rafii M., Lebarron M., Chang J., Ball R.O., Kong D., Xu L., Elango R., Courtney-Martin G. (2021). Dietary leucine requirement of older men and women is higher than current recommendations. Am. J. Clin. Nutr..

[41] Casperson S.L., Sheffield-Moore M., Hewlings S.J., Paddon-Jones D. (2012). Leucine supplementation chronically improves muscle protein synthesis in older adults consuming the RDA for protein. Clin. Nutr..

[42] Voulgaridou G., Papadopoulou S.D., Spanoudaki M., Kondyli F.S., Alexandropoulou I., Michailidou S., Zarogoulidis P., Matthaios D., Giannakidis D., Romanidou M. (2023). Increasing Muscle Mass in Elders through Diet and Exercise: A Literature Review of Recent RCTs. Foods.

[43] Bauer J.M., Verlaan S., Bautmans I., Brandt K., Donini L.M., Maggio M., McMurdo M.E.T., Mets T., Seal C., Wijers S.L. (2015). Effects of a Vitamin D and Leucine-Enriched Whey Protein Nutritional Supplement on Measures of Sarcopenia in Older Adults, the PROVIDE Study: A Randomized, Double-Blind, Placebo-Controlled Trial. J. Am. Med. Dir. Assoc..

[44] Lee E.-J., Neppl R.L. (2021). Influence of Age on Skeletal Muscle Hypertrophy and Atrophy Signaling: Established Paradigms and Unexpected Links. Genes.

[45] Barkoukis H. (2016). Muscle Building and Maintenance in the Elderly: The Use of Protein. Curr. Nutr. Rep..

[46] Chen S., Lin X., Ma J., Li M., Chen Y., Fang A., Zhu H. (2023). Dietary protein intake and changes in muscle mass measurements in community-dwelling middle-aged and older adults: A prospective cohort study. Clin. Nutr..

[47] Yuan M., Pickering R.T., Bradlee M.L., Mustafa J., Singer M.R., Moore L.L. (2021). Animal protein intake reduces risk of functional impairment and strength loss in older adults. Clin. Nutr..

[48] Wall B.T., Gorissen S.H., Pennings B., Koopman R., Groen B.B.L., Verdijk L.B., Van Loon L.J.C. (2015). Aging Is Accompanied by a Blunted Muscle Protein Synthetic Response to Protein Ingestion. PLoS ONE.

[49] Tezze C., Sandri M., Tessari P. (2023). Anabolic Resistance in the Pathogenesis of Sarcopenia in the Elderly: Role of Nutrition and Exercise in Young and Old People. Nutrients.

[50] Trommelen J., Van Lieshout G.A.A., Nyakayiru J., Holwerda A.M., Smeets J.S.J., Hendriks F.K., Van Kranenburg J.M.X., Zorenc A.H., Senden J.M., Goessens J.P.B. (2023). The anabolic response to protein ingestion during recovery from exercise has no upper limit in magnitude and duration in vivo in humans. Cell Rep. Med..

[51] Moore D.R., Churchward-Venne T.A., Witard O., Breen L., Burd N.A., Tipton K.D., Phillips S.M. (2015). Protein Ingestion to Stimulate Myofibrillar Protein Synthesis Requires Greater Relative Protein Intakes in Healthy Older Versus Younger Men. J. Gerontol. Ser. A.

[52] Van Der Heijden I., West S., Monteyne A.J., Finnigan T.J.A., Abdelrahman D.R., Murton A.J., Stephens F.B., Wall B.T. (2024). Ingestion of a variety of non-animal-derived dietary protein sources results in diverse postprandial plasma amino acid responses which differ between young and older adults. Br. J. Nutr..

[53] Reitelseder S., Bülow J., Holm L. (2021). Divergent Anabolic Response to Exercise in Young and Older Adult Men-Dependency on Time Frame of Measurement. J. Gerontol. Ser. A.

[54] Yu X., Sun S., Zhang S., Hao Q., Zhu B., Teng Y., Long Q., Li S., Lv Y., Yue Q. (2022). A pooled analysis of the association between sarcopenia and osteoporosis. Medicine.

[55] Coletta G., Phillips S.M. (2023). An elusive consensus definition of sarcopenia impedes research and clinical treatment: A narrative review. Ageing Res. Rev..

[56] Wiedmer P., Jung T., Castro J.P., Pomatto L.C.D., Sun P.Y., Davies K.J.A., Grune T. (2021). Sarcopenia—Molecular mechanisms and open questions. Ageing Res. Rev..

[57] Liu Z., Liang Q., Ren Y., Guo C., Ge X., Wang L., Cheng Q., Luo P., Zhang Y., Han X. (2023). Immunosenescence: Molecular mechanisms and diseases. Signal Transduct. Target. Ther..

[58] Lian J., Yue Y., Yu W., Zhang Y. (2020). Immunosenescence: A key player in cancer development. J. Hematol. Oncol.J Hematol Oncol.

[59] Rodrigues L.P., Teixeira V.R., Alencar-Silva T., Simonassi-Paiva B., Pereira R.W., Pogue R., Carvalho J.L. (2021). Hallmarks of aging and immunosenescence: Connecting the dots. Cytokine Growth Factor Rev..

[60] Ohtani N. (2022). The roles and mechanisms of senescence-associated secretory phenotype (SASP): Can it be controlled by senolysis?. Inflamm. Regen..

[61] Wang Y., Dong C., Han Y., Gu Z., Sun C. (2022). Immunosenescence, aging and successful aging. Front. Immunol..

[62] Iddir M., Brito A., Dingeo G., Fernandez Del Campo S.S., Samouda H., La Frano M.R., Bohn T. (2020). Strengthening the Immune System and Reducing Inflammation and Oxidative Stress through Diet and Nutrition: Considerations during the COVID-19 Crisis. Nutrients.

[63] Fulop T., Wagner J.R., Khalil A., Weber J., Trottier L., Payette H. (1999). Relationship Between the Response to Influenza Vaccination and the Nutritional Status in Institutionalized Elderly Subjects. J. Gerontol. A Biol. Sci. Med. Sci..

[64] Langsetmo L., Harrison S., Jonnalagadda S., Pereira S.L., Shikany J.M., Farsijani S., Lane N.E., Cauley J.A., Stone K., Cawthon P.M. (2020). Low Protein Intake Irrespective of Source is Associated with Higher Mortality Among Older Community-Dwelling Men. J. Nutr. Health Aging.

[65] Nunes-Cabaço H., Moita D., Rôla C., Mendes A.M., Prudêncio M. (2022). Impact of Dietary Protein Restriction on the Immunogenicity and Efficacy of Whole-Sporozoite Malaria Vaccination. Front. Immunol..

[66] Hoang T., Agger E.M., Cassidy J.P., Christensen J.P., Andersen P. (2015). Protein Energy Malnutrition during Vaccination Has Limited Influence on Vaccine Efficacy but Abolishes Immunity if Administered during Mycobacterium tuberculosis Infection. Infect. Immun..

[67] Collins N. (2020). Dietary Regulation of Memory T Cells. Int. J. Mol. Sci..

[68] Kelly B., Pearce E.L. (2020). Amino Assets: How Amino Acids Support Immunity. Cell Metab..

[69] Yang L., Chu Z., Liu M., Zou Q., Li J., Liu Q., Wang Y., Wang T., Xiang J., Wang B. (2023). Amino acid metabolism in immune cells: Essential regulators of the effector functions, and promising opportunities to enhance cancer immunotherapy. J. Hematol. Oncol. J. Hematol. Oncol..

[70] Li P., Wu G. (2022). Important roles of amino acids in immune responses. Br. J. Nutr..

[71] Abnousian A., Vasquez J., Sasaninia K., Kelley M., Venketaraman V. (2023). Glutathione Modulates Efficacious Changes in the Immune Response against Tuberculosis. Biomedicines.

[72] Ko G.-J., Rhee C.M., Kalantar-Zadeh K., Joshi S. (2020). The Effects of High-Protein Diets on Kidney Health and Longevity. J. Am. Soc. Nephrol..

[73] Jhee J.H., Kee Y.K., Park S., Kim H., Park J.T., Han S.H., Kang S.-W., Yoo T.-H. (2019). High-protein diet with renal hyperfiltration is associated with rapid decline rate of renal function: A community-based prospective cohort study. Nephrol. Dial. Transplant..

[74] Oh S.W., Yang J.H., Kim M.-G., Cho W.Y., Jo S.K. (2020). Renal hyperfiltration as a risk factor for chronic kidney disease: A health checkup cohort study. PLoS ONE.

[75] Tang Y., Jiang J., Zhao Y., Du D. (2024). Aging and chronic kidney disease: Epidemiology, therapy, management and the role of immunity. Clin. Kidney J..

[76] Hahn D., Hodson E.M., Fouque D. (2018). Low protein diets for non-diabetic adults with chronic kidney disease. Cochrane Database Syst. Rev..

[77] Ko G.-J., Kalantar-Zadeh K. (2021). How important is dietary management in chronic kidney disease progression? A role for low protein diets. Korean J. Intern. Med..

[78] Pradhan N., Dobre M. (2023). Emerging Preventive Strategies in Chronic Kidney Disease: Recent Evidence and Gaps in Knowledge. Curr. Atheroscler. Rep..

[79] Ikizler T.A., Burrowes J.D., Byham-Gray L.D., Campbell K.L., Carrero J.-J., Chan W., Fouque D., Friedman A.N., Ghaddar S., Goldstein-Fuchs D.J. (2020). KDOQI Clinical Practice Guideline for Nutrition in CKD: 2020 Update. Am. J. Kidney Dis..

[80] Garneata L., Mocanu C.-A., Mircescu G. (2024). Low-Protein Diets Could Be Effective and Safe in Elderly Patients with Advanced Diabetic Kidney Disease. Nutrients.

[81] Carballo-Casla A., Avesani C.M., Beridze G., Ortolá R., García-Esquinas E., Lopez-Garcia E., Dai L., Dunk M.M., Stenvinkel P., Lindholm B. (2024). Protein Intake and Mortality in Older Adults with Chronic Kidney Disease. JAMA Netw. Open.

[82] Rautakallio-Järvinen P., Kunvik S., Laaksonen M., Fogelholm L., Nykänen I., Schwab U. (2024). Cost-effectiveness of protein-rich meals and snacks for increasing protein intake in older adults. J. Nutr. Health Aging.

[83] Papanikolaou Y., Fulgoni V.L. (2020). Eggs Are Cost-Efficient in Delivering Several Shortfall Nutrients in the American Diet: A Cost-Analysis in Children and Adults. Nutrients.

[84] Hess J.M., Cifelli C.J., Agarwal S., Fulgoni V.L. (2019). Comparing the cost of essential nutrients from different food sources in the American diet using NHANES 2011–2014. Nutr. J..

[85] Rehm C.D., Monsivais P., Drewnowski A. (2011). The quality and monetary value of diets consumed by adults in the United States. Am. J. Clin. Nutr..

[86] Dismore L., Sayer A., Robinson S. (2024). Exploring the experience of appetite loss in older age: Insights from a qualitative study. BMC Geriatr..

[87] Warner J., Stocker R., Brandt K., Crabtree D.R., Ormond L., Stevenson E., Holliday A. (2024). Appetite, food intake, and gut hormone responses to glycomacropeptide protein ingestion in older adults: A feasibility, acceptability, and pilot study. Appetite.

[88] Hendriks-Hartensveld A.E.M., Havermans R.C., Nederkoorn C., Van Den Heuvel E. (2024). Exploring within-meal variety to promote appeal of home-cooked meals in older adults. Appetite.

[89] Teodoro M.A., Silva W.R.D., Spexoto M.C.B., Silva Júnior S.I.D. (2024). Factors of food choice and nutritional intake of Brazilian older adults according sociodemographic and health characteristics. Appetite.

[90] Kotronia E., Brown H., Papacosta A.O., Lennon L.T., Weyant R.J., Whincup P.H., Wannamethee S.G., Ramsay S.E. (2021). Poor oral health and the association with diet quality and intake in older people in two studies in the UK and USA. Br. J. Nutr..

[91] Zhang Y., Leveille S.G., Shi L. (2022). Multiple Chronic Diseases Associated with Tooth Loss Among the US Adult Population. Front. Big Data.

[92] Centers for Disease Control and Prevention (2019). Oral Health Surveillance Report: Trends in Dental Caries and Sealants, Tooth Retention, and Edentulism, United States, 1999–2004 and 2011–2016.

[93] Kimble R., Papacosta A.O., Lennon L.T., Whincup P.H., Weyant R.J., Mathers J.C., Wannamethee S.G., Ramsay S.E. (2023). The Relationship of Oral Health with Progression of Physical Frailty among Older Adults: A Longitudinal Study Composed of Two Cohorts of Older Adults from the United Kingdom and United States. J. Am. Med. Dir. Assoc..

[94] Mendonça N., Granic A., Mathers J.C., Hill T.R., Siervo M., Adamson A.J., Jagger C. (2018). Prevalence and determinants of low protein intake in very old adults: Insights from the Newcastle 85+ Study. Eur. J. Nutr..

[95] Albani V., Nishio K., Ito T., Kotronia E., Moynihan P., Robinson L., Hanratty B., Kingston A., Abe Y., Takayama M. (2021). Associations of poor oral health with frailty and physical functioning in the oldest old: Results from two studies in England and Japan. BMC Geriatr..

[96] Chan A.K.Y., Tsang Y.C., Jiang C.M., Leung K.C.M., Lo E.C.M., Chu C.H. (2023). Diet, Nutrition, and Oral Health in Older Adults: A Review of the Literature. Dent. J..

[97] Agergaard J., Justesen T.E.H., Jespersen S.E., Tagmose Thomsen T., Holm L., Van Hall G. (2023). Even or skewed dietary protein distribution is reflected in the whole-body protein net-balance in healthy older adults: A randomized controlled trial. Clin. Nutr..

[98] Verreijen A.M., Van Den Helder J., Streppel M.T., Rotteveel I., Heman D., Van Dronkelaar C., Memelink R.G., Engberink M.F., Visser M., Tieland M. (2021). A higher protein intake at breakfast and lunch is associated with a higher total daily protein intake in older adults: A post-hoc cross-sectional analysis of four randomised controlled trials. J. Hum. Nutr. Diet..

[99] Souza L.B.D., Martins K.A., Bomfim R.A. (2022). Inadequate distribution of dietary protein and muscle mass in older adults. Geriatr. Gerontol. Aging.

[100] Hiol A.N., Von Hurst P.R., Conlon C.A., Beck K.L. (2023). Associations of protein intake, sources and distribution on muscle strength in community-dwelling older adults living in Auckland, New Zealand. J. Nutr. Sci..

[101] Koopmans L., Van Oppenraaij S., Heijmans M.W.F., Verlaan S., Schoufour J.D., Ten Haaf D.S.M., Van Der Avoort C.M.T., Van Den Helder J., Memelink R., Verreijen A. (2025). Dietary protein intake, protein sources & distribution patterns in community-dwelling older adults: A harmonized analysis of eight studies. Clin. Nutr..

[102] Famularo P. (2023). Protein Requirements for Older Adults: What Are the Current Recommendations for Intake?. Caring Ages.

[103] Paddon-Jones D., Leidy H. (2014). Dietary protein and muscle in older persons. Curr. Opin. Clin. Nutr. Metab. Care.

[104] Justesen T.E.H., Jespersen S.E., Tagmose Thomsen T., Holm L., Van Hall G., Agergaard J. (2022). Comparing Even with Skewed Dietary Protein Distribution Shows No Difference in Muscle Protein Synthesis or Amino Acid Utilization in Healthy Older Individuals: A Randomized Controlled Trial. Nutrients.

[105] Jespersen S.E., Agergaard J. (2021). Evenness of dietary protein distribution is associated with higher muscle mass but not muscle strength or protein turnover in healthy adults: A systematic review. Eur. J. Nutr..

[106] Hudson J., Bergia R., Campbell W. (2020). Protein Distribution and Muscle-Related Outcomes: Does the Evidence Support the Concept?. Nutrients.

[107] Hettiarachchi J., Reijnierse E.M., Kew N., Fetterplace K., Tan S.-Y., Maier A.B. (2024). The effect of dose, frequency, and timing of protein supplementation on muscle mass in older adults: A systematic review and meta-analysis. Ageing Res. Rev..

[108] Campbell W.W., Deutz N.E.P., Volpi E., Apovian C.M. (2023). Nutritional Interventions: Dietary Protein Needs and Influences on Skeletal Muscle of Older Adults. J. Gerontol. Ser. A.

[109] Holwerda A.M., Trommelen J., Kouw I.W.K., Senden J.M., Goessens J.P.B., Van Kranenburg J., Gijsen A.P., Verdijk L.B., Van Loon L.J.C. (2021). Exercise Plus Presleep Protein Ingestion Increases Overnight Muscle Connective Tissue Protein Synthesis Rates in Healthy Older Men. Int. J. Sport Nutr. Exerc. Metab..

[110] Trommelen J., Van Loon L. (2016). Pre-Sleep Protein Ingestion to Improve the Skeletal Muscle Adaptive Response to Exercise Training. Nutrients.

[111] Weijzen M.E.G., Kohlen M., Monsegue A., Houtvast D.C.J., Nyakayiru J., Beijer S., Geerlings P., Verdijk L.B., Van Loon L.J.C. (2024). Access to a pre-sleep protein snack increases daily energy and protein intake in surgical hospitalized patients. Clin. Nutr..

[112] Res P.T., Groen B., Pennings B., Beelen M., Wallis G.A., Gijsen A.P., Senden J.M.G., Van Loon L.J.C. (2012). Protein Ingestion before Sleep Improves Postexercise Overnight Recovery. Med. Sci. Sports Exerc..

[113] Trommelen J., Van Lieshout G.A.A., Pabla P., Nyakayiru J., Hendriks F.K., Senden J.M., Goessens J.P.B., Van Kranenburg J.M.X., Gijsen A.P., Verdijk L.B. (2023). Pre-sleep Protein Ingestion Increases Mitochondrial Protein Synthesis Rates During Overnight Recovery from Endurance Exercise: A Randomized Controlled Trial. Sports Med..

[114] Höglund E., Ekman S., Stuhr-Olsson G., Lundgren C., Albinsson B., Signäs M., Karlsson C., Rothenberg E., Wendin K. (2018). A meal concept designed for older adults—Small, enriched meals including dessert. Food Nutr. Res..

[115] Delsoglio M., Griffen C., Syed R., Cookson T., Saliba H., Vowles A., Davies S., Willey N., Thomas J., Millen N. (2023). A multi-center prospective study of plant-based nutritional support in adult community-based patients at risk of disease-related malnutrition. Front. Nutr..

[116] Jun S.-H., Lee J.W., Shin W.-K., Lee S.-Y., Kim Y. (2023). Association between plant protein intake and grip strength in Koreans aged 50 years or older: Korea National Health and Nutrition Examination Survey 2016–2018. Nutr. Res. Pract..

[117] Chen L., Arai H., Assantachai P., Akishita M., Chew S.T.H., Dumlao L.C., Duque G., Woo J. (2022). Roles of nutrition in muscle health of community-dwelling older adults: Evidence-based expert consensus from Asian Working Group for Sarcopenia. J. Cachexia Sarcopenia Muscle.

[118] Habumugisha T., Engebretsen I.M.S., Måren I.E., Kaiser C.W.M., Dierkes J. (2024). Reducing meat and/or dairy consumption in adults: A systematic review and meta-analysis of effects on protein intake, anthropometric values, and body composition. Nutr. Rev..

[119] Pinckaers P.J., Domić J., Petrick H.L., Holwerda A.M., Trommelen J., Hendriks F.K., Houben L.H., Goessens J.P., Van Kranenburg J.M., Senden J.M. (2024). Higher Muscle Protein Synthesis Rates Following Ingestion of an Omnivorous Meal Compared with an Isocaloric and Isonitrogenous Vegan Meal in Healthy, Older Adults. J. Nutr..

[120] McLean R.R., Mangano K.M., Hannan M.T., Kiel D.P., Sahni S. (2016). Dietary Protein Intake Is Protective Against Loss of Grip Strength Among Older Adults in the Framingham Offspring Cohort. J. Gerontol. A. Biol. Sci. Med. Sci..

[121] Lim M.T., Pan B.J., Toh D.W.K., Sutanto C.N., Kim J.E. (2021). Animal Protein versus Plant Protein in Supporting Lean Mass and Muscle Strength: A Systematic Review and Meta-Analysis of Randomized Controlled Trials. Nutrients.

[122] Yeung S.S.Y., Woo J. (2022). Association of Plant Protein Intake with Change in Physical Performance in Chinese Community-Dwelling Older Adults. Nutrients.

[123] Du Q., Lu Y., Hu F., Feng X., Zhang Y., Li S., Zhang C., Zhang H., Zeng Y., Yao Y. (2023). Dietary diversity and possible sarcopenia among older people in China: A nationwide population-based study. Front. Nutr..

[124] Sakaguchi Y., Kaimori J.-Y., Isaka Y. (2023). Plant-Dominant Low Protein Diet: A Potential Alternative Dietary Practice for Patients with Chronic Kidney Disease. Nutrients.

